# Contrastive Mask Learning for Self-Supervised 3D Skeleton-Based Action Recognition

**DOI:** 10.3390/s25051521

**Published:** 2025-02-28

**Authors:** Haoyuan Zhang

**Affiliations:** School of Electrical and Information Engineering, North Minzu Univeristy, Yinchuan 750021, China; haoyuan@nmu.edu.cn

**Keywords:** self-supervised learning, contrastive mask learning, 3D skeleton action recognition

## Abstract

In this paper, we propose a contrastive mask learning (CML) method for self-supervised 3D skeleton-based action recognition. Specifically, the mask modeling mechanism is integrated into multi-level contrastive learning with the aim of forming a mutually beneficial learning scheme from both contrastive learning and masked skeleton reconstruction. The contrastive objective is extended from an individual skeleton instance to clusters by closing the gap between cluster assignment from different instances of the same category, with the goal of pursuing inter-instance consistency. Compared with previous methods, CML integrates contrastive and masked learning comprehensively and enables intra-/inter-instance consistency pursuit via multi-level contrast, which leads to more discriminative skeleton representation learning. Our extensive evaluation of the challenging NTU RGB+D and PKU-MMD benchmarks demonstrates that representations learned via CML exhibit superior discriminability, consistently outperforming state-of-the-art methods in terms of action recognition accuracy.

## 1. Introduction

Human action recognition has emerged as an active research domain in computer vision. Three-dimensional skeleton representations have garnered increasing attention in modeling human motion dynamics, due to their lightweight architecture, privacy-preserving characteristics, and robustness in complex environments [1]. Although the majority of existing skeleton-based recognition approaches [2,3,4,5] were predominantly developed under full supervision, their reliance on substantial quantities of annotated skeleton data—the acquisition of which entails significant costs and operational challenges—has motivated recent explorations into self-supervised representation learning paradigms. Existing self-supervised approaches can be categorized into two principal strands: pretext task-based approaches employing reconstruction [6] or jigsaw puzzle-solving [7] and contrastive learning frameworks [8,9,10,11,12,13,14] that perform instance discrimination in latent spaces. These approaches exhibit inherent limitations, as their effectiveness is contingent upon either the manual design of pretext tasks with limited generalizability or the requirement for sophisticated contrastive pair construction.

Although contrastive learning-based approaches have dominated the field of 3D skeleton representation learning for some time, several issues exist and impede further progress. To be more specific, the contrastive objective of the general contrastive learning methods involves skeleton instances, and it is difficult to explore high-level relations among the data; thus, a discriminative feature space cannot be formed among all skeleton instances. Furthermore, different augmented versions from the same skeleton instance are treated as the only positives and pulled close, ignoring the the inter-instance consistency of current skeleton instances and the non-self skeleton instances with the same class, limiting the representative ability for intra-class diversity. Most works have utilized masked self-reconstruction of human joints as the pretext task, and the power of mask skeleton modeling combined with contrastive learning warrants further exploration. To solve the above issues, the mask learning mechanism was integrated into contrastive learning in this study, and a mutually beneficial learning scheme was constructed from both contrastive learning and masked skeleton reconstruction. Then, the contrastive objective was extended from individual instances by introducing cluster-level contrast to construct the multi-level contrast paradigm. Clustering contrast involves relaxing the instance discrimination problem by discriminating between groups of skeleton instances with similar features. The contrastive mask learning method described above enables intra-/inter-instance consistency pursuit and further boosts representation learning.

To this end, in this paper, a contrastive mask learning (CML) paradigm is proposed for self-supervised skeleton 3D action representation learning. CML consists of three learning components, the mask learning branch and the instance-level and cluster-level comparisons. In the case of instance-level contrast, a Siamese structure with a student and a teacher branch that follows BYOL [15] is leveraged. The mask learning branch is trained to predict the original skeleton sequence for skeleton reconstruction and provides novel contrast views for contrastive learning. The student network is trained to match the teacher network in terms of the similarity of the augmentations from the same instance to learn the intra-instance consistency, and the gradients of the student branch guide the mask learning with high-level semantics. In cluster-level contrast, the cluster assignments are utilized as contrastive targets during training, and cluster assignments of different instances with the same category are enforced as consistent, such that the inter-instance consistency can be learned. Specifically, in the teacher branch, the most similar skeleton instances in a contextual queue are searched as the extra positive of the current instance to match the cluster assignments, aimed at enriching intra-class diversity, and representation learning is further boosted. The experimental results on NTU RGB+D [16] and PKU-MMD [17] datasets verify the effectiveness of proposed contrastive mask learning.

To summarize, our contributions include the following:

- We propose a novel self-supervised skeleton 3D skeleton action representation learning scheme, the contrastive mask learning (CML) paradigm.
- The mask learning mechanism is introduced into contrastive learning, forming a mutually beneficial learning scheme from both contrastive learning and masked skeleton reconstruction.
- A multi-level contrast paradigm is leveraged to learn both inter-instance and intra-instance consistency of the skeleton action.
- The proposed learning paradigm was evaluated on NTU RGB+D [16] and PKU-MMD [17] datasets, achieving promising results.

## 2. Related Works

### 2.1. Self-Supervised Contrastive Learning

Contrastive learning approaches have emerged as a highly successful paradigm within self-supervised learning approaches. Originating from noise-contrastive estimation (NCE) [18], which employs noise differentiation for latent distribution estimation, this framework was significantly advanced by Contrastive Predictive Coding (CPC) [19] through its extension to info-NCE for image representation learning. A critical limitation of these methods lies in their restricted access to negative instances. To address this, the memory-bank mechanism [20] was introduced. This mechanism stores historical random representations as negative samples, each treated as distinct classes. Subsequent innovations include Momentum Contrast (MoCo) [21], which enhances the memory bank with a dynamic dictionary and incorporates a momentum-updated encoder to improve representation learning. The SimCLR framework [22] further advanced the field by utilizing large batch sizes to generate negative instances, eliminating the need for a memory bank, while demonstrating the impact of various components on contrastive learning performance. However, these methods exhibit two fundamental limitations: firstly, their contrastive objectives focus solely on individual instances, and they fail to establish a discriminative feature space across all instances; secondly, they treat different augmentations of the same instance as exclusive positives while considering all other instances, including those from the same category, as negatives to be repelled.

Recent developments have introduced negative-sample-free approaches to overcome these limitations. The SimSiam method [23] offers a simplified Siamese architecture that eliminates requirements, such as large batch sizes, employing stop-gradient operations to prevent collapse while achieving effective representation learning. The Barlow Twins approach [24] represents a twin-network structure with a novel objective function based on cross-correlation matrix analysis between twin encoder outputs. The BYOL method [15] comprises an online-target framework where one network predicts the representation of another. Despite these advancements, current approaches remain focused on contrasting individual instances and treating different augmentations of the same instance as exclusive positives, thereby neglecting inter-instance consistency learning within the same class and failing to explore higher-level data relationships.

### 2.2. Self-Supervised 3D Action Recognition

While self-supervised learning for video-based action representations has achieved notable progress [25,26], research on skeleton-based 3D action recognition remains comparatively underexplored. Current methodologies exhibit diverse technical innovations: LongT GAN [6] is a pioneering auto-encoder GAN architecture that reconstructs raw skeleton sequences through adversarial training with bidirectional LSTM encoders. The P&C framework [9] offers a novel encoder–decoder paradigm where deliberately weakened encoder capacity (achieved via layer pruning) forces the model to learn compressed yet discriminative spatiotemporal features. In the field of temporal modeling, ASCAL [8] enhances LSTM networks through integrating momentum-based weight updates and a dynamically refreshed memory bank that stores historical skeleton motion patterns. In multi-modal learning, MS2L [7] synergizes contrastive instance discrimination with three handcrafted pretext tasks (joint masking, motion prediction, and sequence reversal), while CrosSCLR [10] constructs cross-view positive pairs by projecting skeleton sequences into multiple camera coordinate systems for contrastive alignment. ISC [27] further extends this concept through cross-contrastive learning between heterogeneous skeleton representations (e.g., joint coordinates vs. bone vectors).

Recent advances demonstrate several evolutionary directions. CPM [28] addresses positive sample scarcity though mining semantically similar "non-self" instances across batches as auxiliary positives. CSCLR [29] improves latent space discriminability through creating hard negative samples through inter-stream feature mixing between motion and joint streams. HiCo [30] employs a pyramidal sequence-to-sequence encoder with learnable downsampling modules to hierarchically capture action semantics at frame, segment, and sequence levels. Regarding view invariance, ViA [13] implements skeletal motion retargeting across virtual viewpoints using geometric transformations, enforcing consistency through asymmetric contrastive learning. AML [14] uses dual attention mechanisms (spatial joint importance scoring and temporal saliency detection) to guide feature masking, where models must recognize actions from partially obscured skeleton sequences through contrastive comparisons with unmasked counterparts. Despite these technical advancements, prevailing methods face two persistent challenges: over-reliance on conventional contrastive learning that inherits problematic negative sampling practices (e.g., treating same-class instances as negatives) and insufficient modeling of inter-instance relationships, particularly for learning category-level consistency across semantically similar actions. These limitations fundamentally constrain their capacity to learn transferable representations for downstream recognition tasks.

## 3. Proposed Method

Although successes have been achieved in 3D skeleton action recognition, self-supervised skeleton representation learning still requires further exploration. We expect to extend the contrast objective and take advantage of mask skeleton modeling to form a mutual learning scheme by focusing on the issues of the insufficient learning of single-level comparison in sole contrastive learning, thus developing the contrastive mask learning (CML) paradigm for 3D skeleton action representation.

### 3.1. Basic Framework

**Figure 1:**
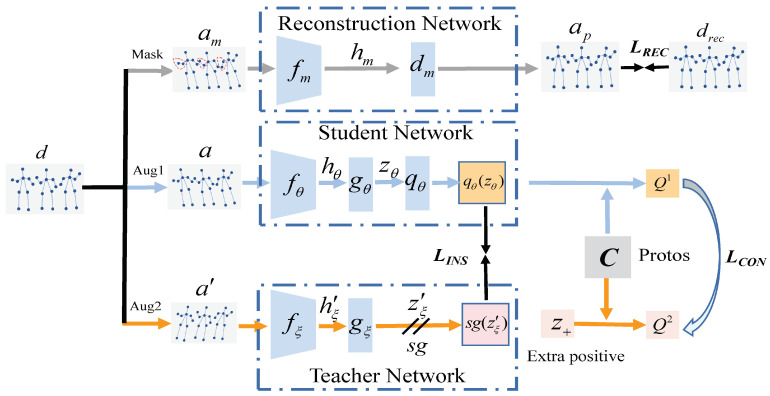
Overview of the CML framework.

As illustrated in Figure 1, our CML framework for self-supervised skeleton action recognition comprises three core components, which are detailed below. Given an input skeleton sequence $d\in R^{C\times T\times V}$ with *C* coordinate channels, *T* temporal frames, and *V* body joints, augmented views *a* and $a^{\prime }$ are generated through a hybrid augmentation strategy. Spatial augmentation: shear transformation is employed to apply a channel-wise linear transformation matrix to skew joint coordinates at randomized angles, simulating biomechanical variations. Temporal augmentation: symmetric frame padding followed by randomized cropping [8,10] preserves temporal coherence while introducing duration variability. Spatiotemporal fusion: CutMix [31] synthesizes hybrid sequences by cross-pasting spatiotemporal regions between pairs, enhancing robustness to partial occlusion.

Triple encoders with an identical network structure are then used to produce representations of the masked and augmented skeleton sequence [15]. The first branch of the triple encoders is referred to as the reconstruction network $f_{m}$, which generates the masked skeleton sequence and reconstructs the original skeleton. The second branch is the student network, and the third branch called the teacher network $f_{\xi }$ is designed to produce targets for the student branch to predict. The parameters of the reconstruction branch are updated via the gradients from the student branch, and the teacher parameters ξ are updated with a slowly exponential moving average of the student parameters θ. More specifically, after each training step, the ξ is updated as follows with the target decay rate $\tau \in \left[0,\;1\right]$:

(1) $$\tau \xi +\left(1-\tau \right)\theta \to \xi$$

The ST-GCN [32] architecture constructs spatiotemporal graphs where nodes represent joints, and edges encode both anatomical connections (spatial) and inter-frame motion (temporal). Each GCN layer alternates between spatial graph convolution (capturing joint correlations) and temporal convolution (modeling motion dynamics). Triple encoders consisting of several GCN layers are used to embed three batches of masked and augmented skeleton sequences $a_{m}$, *a*, and $a^{\prime }$ into latent space. In each layer, to alternatively encode the human pose in the spatial dimension and the joint’s motion in the temporal dimension, a spatial graph convolution is followed by a temporal convolution.

After the triple encoders are employed, a decoder consisting of one GCN layer is leveraged in the reconstruction network to output the reconstructed skeleton $a_{p}$, while the projection MLP is attached in the student and teacher branches to project the hidden vector, $z_{\theta }=g_{\theta }\left(h_{\theta }\right)$, $z_{\xi }^{\prime }=g_{\xi }\left(h_{\xi }^{\prime }\right)$, where $z_{m}$, $z_{\theta }$, and $z_{\xi }^{\prime }$ are assumed to be mean-centered along the batch dimension so that each unit has 0 mean output over the batch. The prediction MLP $q_{\theta }$ with the same architecture as the $h_{\theta }$ is then appended to the student branch to produce the prediction $q_{\theta }\left(z_{\theta }\right)$, while the stop-gradient operation is used in the teacher branch with the output $sg(z_{\xi }^{\prime })$.

### 3.2. Masked Skeleton Modeling

In the reconstruction branch, the original skeleton is first masked to acquire the masked version. Then, the masked skeleton is encoded by the mask encoder $f_{m}$ to obtain the corresponding skeleton features. After the encoder, a decoder $d_{m}$ is attached, and the encoded features are fed to $d_{m}$ to generate the reconstructed skeleton $d_{rec}$ so that the masked part of the original skeleton can be predicted. Specifically, the body-part masking strategy is leveraged instead of masking individual joints for the purpose of further enriching the learned skeleton representations from a higher level. The skeleton is divided into five parts: the torso, left hand, left arm, right leg, and right arm. The same parts from the identical clip are all masked.

Then, the mean squared error (MSE) loss is optimized between the reconstructed skeleton and original sequence:

(2) $$L_{REC}\overset{\Delta }{=}{∥d_{m}-d_{rec}∥}_{2}^{2}$$

### 3.3. Multi-Level Contrast

In CML, the instance-level contrast is first conducted to learn the intra-instance consistency through bringing the different augmentations of the same instance close. To be more specific, the predictions $q_{\theta }\left(z_{\theta }\right)$ and target projections $sg(z_{\xi }^{\prime })$ are first $l_{2}$ normalized. Then, the MSE loss between them is leveraged as the training objective to draw a pair of augmentations from an identical instance in the student and teacher network. The teacher network produces the regression targets to train the student branch:

(3) $$L_{INS}\overset{\Delta }{=}{∥{\bar{q}}_{\theta }\left(z_{\theta }\right)-{{\bar{z}}^{\prime }}_{\xi }∥}_{2}^{2}=2-2\cdot \frac{\langle q_{\theta }\left(z_{\theta }\right),{z^{\prime }}_{\xi }\rangle }{{∥q_{\theta }\left(z_{\theta }\right)∥}_{2}\cdot {∥{z^{\prime }}_{\xi }∥}_{2}}$$

The loss is symmetrized by separately feeding $x^{\prime }$ to the student network and *x* to the teacher network to compute ${\tilde{L}}_{INS}$, and the final loss is as follows:

(4) $$L_{INS}^{\prime }=L_{INS}+{\tilde{L}}_{INS}$$

A stochastic optimization step is performed to minimize ${\tilde{L}}_{mse}$ at each training step with respect to θ only because the stop-gradient operation is used in the teacher network.

After the instance-level contrast, the contrast objective is extended from the individual instance to the cluster with the aim of learning the inter-instance consistency from a higher level. The predictions $q_{\theta }\left(z_{\theta }\right)$ and target projections $sg(z_{\xi }^{\prime })$ are further leveraged for a cluster-level comparison, forming a multi-level contrast paradigm.

Instead of directly bringing the cluster assignment of $q_{\theta }\left(z_{\theta }\right)$ and $sg(z_{\xi }^{\prime })$ together [33], which still leads to focus on the intra-instance consistency and a lack of intra-class diversity, we propose to substitute $sg(z_{\xi }^{\prime })$ with the extra positive instance $z_{+}$. To be more specific, $z_{+}$ is from the reconstruction branch where the reconstruction output $a_{p}$ is utilized. The cluster assignment of $q_{\theta }\left(z_{\theta }\right)$ and $z_{+}$ is then matched.

The computation of clusters is inspired by SwAV [33]. A set of randomly initialized prototypes that are updated in each batch are stored, with $C=\left\{c_{1},\cdots ,c_{K}\right\}\in R^{d\times K}$, where *K* is the number of clusters, and $d_{p}$ is the dimension of the prototypes that remains the same as $q_{\theta }\left(z_{\theta }\right)$ and $z_{+}$. Then, the cluster assignments are computed within a batch. To be specific, given *B* feature vectors $Z^{1}=sg\left({Z^{\prime }}_{\xi }\right)=\left[z_{1}^{1},\cdots ,z_{B}^{1}\right]$ (*B* is batch size); $Z^{2}=Z_{+}=\left[z_{1}^{2},\cdots ,z_{B}^{2}\right]$; and *K* prototypes $C=\left[c_{1},\cdots ,c_{K}\right]$, the probability of each instance belonging to a specific cluster is first computed by measuring the similarity between the skeleton embedding $z_{i}^{1}$ and the prototype $c_{j}$,

(5) $$p_{i,j}^{1}=\frac{\exp\left(\frac{1}{\tau }\langle {\bar{z}}_{i}^{1},{\bar{c}}_{j}\rangle \right)}{\sum_{j^{\prime }}\exp\left(\frac{1}{\tau }\langle {\bar{z}}_{i}^{1},{\bar{c}}_{j^{\prime }}\rangle \right)}$$

where ${\bar{z}}_{i}^{1}$ and ${\bar{c}}_{j}$ are the $l_{2}$ normalization of $z_{i}^{1}$ and $c_{j}$, and τ is a temperature parameter. Then, the cluster assignment $Q^{2}$ for $Z^{2}$ is obtained via mapping $z_{i}^{2}$ to $c_{j}$. By using a fast version of the Sinkhorn–Knopp algorithm [33], an equi-partition constraint (the prototypes equally partition the data) can be enforced to avoid degenerate solutions. Then, $Q^{2}$ can be computed through the student branch for each mini-batch and via solving the following optimization problem:

(6) $$Q^{2}=\max_{Q\in Q}Tr\left(Q^{T}C^{T}Z^{2}\right)+\varepsilon H\left(Q\right)$$

where $Q=\left\{q_{1},\cdots ,q_{B}\right\}\in R_{+}^{K\times B}$, and Q is the transportation polytope defined by

(7) $$Q=\left\{Q\in R_{+}^{K\times B}|Q1_{B}=\frac{1}{K}1_{K},Q^{T}1_{K}=\frac{1}{B}1_{B}\right\}$$

where $1_{K}$ is a vector of ones with dimension *K*, *H* is the entropy function, $H\left(Q\right)=-\sum_{i,j}Q_{i,j}\log Q_{i,j}$, and ε is a parameter that controls the smoothness of the mapping.

Then, to maintain consistency between the clusterings of $Z^{1}$ and $Z^{2}$, a loss which brings the probabilities $p_{i,j}^{1}$ and the cluster assignment $q_{i,j}^{2}$ close is given as follows:

(8) $$L_{CON}=-\frac{1}{2B}\sum_{i=1}^{B}\sum_{j=1}^{K}q_{ij}^{2}\log p_{ij}^{1}$$

The loss is also symmetrized to compute ${\tilde{L}}_{CON}$ with probabilities $p_{i,j}^{2}$ and the cluster assignment $q_{i,j}^{1}$ for swapped prediction. Thus, the final loss is as follows:

(9) $$L_{CON}^{\prime }=L_{CON}+{\tilde{L}}_{CON}$$

This loss function is jointly minimized with respect to the prototypes *C* and the parameters θ of the student encoder $f_{\theta }$.

### 3.4. Mutual Learning Scheme

To enable contrastive learning and masked skeleton modeling to benefit each other, inspired by CMAE [34], we leverage a mutual learning strategy. To be more specific, the gradients of the student branch are propagated to update the reconstructed decoder, which provides high-level semantic guidance for the skeleton reconstruction. This not only boosts masked reconstruction learning but also enriches positive samples with better prediction. Furthermore, the masked skeleton $a_{m}$ is leveraged as another augmentation in the instance-level learning branch, and the reconstruction skeleton $a_{p}$ serves as the extra positive in cluster-level contrastive learning. The masked skeleton strengthens the semantic consistency learning of the model, while the reconstruction skeleton can bring diversity and uncertainty to the model and enable the contrastive learning branch to learn more diverse action patterns. In this way, the skeleton representations learned by the student encoder capture not only holistic features of the input skeleton but also discriminative features, which results in better generalization performance.

Finally, the following loss function is employed:

(10) $$L=\lambda L_{REC}+L_{INS}+L_{CON}$$

where λ is the loss weight.

## 4. Results

To validate the effectiveness of our proposed learning framework, we performed a comprehensive evaluation on two major 3D action recognition benchmarks: NTU RGB+D [16] and PKU-MMD [17]. Our experiments employed self-supervised learning paradigms to enable both quantitative measurements and qualitative assessments.

### 4.1. Dataset

**NTU RGB+D 60**

The NTU-RGB+D 60 dataset [16], recognized as one of the most extensive indoor action recognition resources, contains 56,880 action samples across 60 categories performed by 40 subjects. This multimodal dataset provides 3D skeletal data, RGB video streams, and depth sequences, though our implementation specifically utilized the skeleton modality. Each subject’s motion is captured through 25 anatomical joints represented as 3D coordinates (X, Y, Z) within the camera’s reference frame. The dataset recommends two principal evaluation protocols:

- Cross-Subject (X-Sub): 40,320 training samples are segregated from 16,560 test samples by distinct performer groups, ensuring subject independence between training and evaluation sets.
- Cross-View (X-View): 37,920 training samples from camera Views 2–3 and 18,960 evaluation samples from View 1 are utilized to test viewpoint generalization capabilities.

**NTU RGB+D 120**

The extended NTU-RGB+D 120 dataset [35] expands NTU-60 to 114,480 samples across 120 action classes while maintaining the original data structure. It introduces two enhanced evaluation protocols:

- Cross-Subject (X-Sub): 63,026 training samples and 50,919 test samples are employed with strict subject separation.
- Cross-Setup (X-Set): temporal generalization is challenged through setup ID partitioning using 54,468 training samples and 59,477 test samples.

**PKU-MMD**

For a complementary evaluation, we employed the PKU-MMD dataset [17], containing approximately 20,000 samples across 51 action classes. We specifically analyzed its two subsets, PKU-MMD I (standard) and PKU-MMD II (noise-enhanced), both evaluated under cross-subject protocols to assess robustness against increasing environmental interference.

### 4.2. Implementation Details

**Self-Supervised Pre-Training:** We employed the LARS optimizer [36] with a batch size of 128 for 300 training epochs. The learning rate follows a warm-up strategy, starting from 0 and linearly increasing to 0.75 during the initial 10 epochs then gradually decaying to 0.00075 using a cosine annealing schedule. For data preprocessing, we adopted the methodology from CrosSCLR [10], which involves removing invalid frames and normalizing each skeleton sequence to a fixed length of 50 frames through temporal resizing.

**Linear Evaluation Protocol:** To evaluate the effectiveness of our pre-trained models, we conducted linear classification experiments for action recognition. This evaluation protocol involves training a simple linear classifier (comprising a fully connected layer followed by a softmax activation) for 100 epochs. During this process, the learned representations from the pre-trained models remain frozen, while only the classifier parameters are updated through supervised training.

### 4.3. Experimental Results and Analysis

**Self-Supervised Results**: We comprehensively evaluated our CML framework against existing supervised and self-supervised methods across three benchmark datasets: NTU-60, NTU-120, and PKU-MMD. Following standard evaluation protocols, we report top-1 classification accuracy for all experiments. Unless otherwise specified, our ablation studies and comparative analyses focused on joint data, with “3S” denoting the ensemble results combining joint, bone, and motion modalities. As shown in Table 1, CML demonstrates superior performance over state-of-the-art self-supervised methods and significantly narrows the performance gap with supervised approaches across all datasets. Specifically, CML(3S) achieves 86.8% (X-Sub) and 91.1% (X-View) accuracy on NTU-60, representing improvements of 1.9% and 1.4%, respectively, over AML(3S) [14]. On NTU-120, CML(3S) attains 77.5% (X-Sub) and 78.9% (X-Set) accuracy, outperforming AML(3S) [14] by 2.2% and 1.6%. In PKU-MMD evaluation, CML(3S) achieves 93.5% (PKU-MMD I) and 58.6% (PKU-MMD II) accuracy, demonstrating consistent improvements of 0.2% and 0.6% over AML(3S) [14]. These substantial gains validate CML’s effectiveness for self-supervised, skeleton-based action recognition.

Our semi-supervised evaluation followed a two-stage process: initial pre-training on all training data followed by fine-tuning with limited labeled data (1% and 10% subsets). Table 2 presents comparative results on NTU-60, where CML demonstrates superior performance in low-label regimes. With 1% labeled data, CML achieves 60.2% (X-View) and 59.1% (X-Sub) accuracy, increasing to 83.4% and 80.1%, respectively, with 10% labeled data. These results indicate CML’s enhanced robustness compared to AML [14] when working with limited annotations.

**Semi-Supervised Results**: **Fine-tuned Results**: For a linear evaluation, we conducted self-supervised pre-training followed by training a linear classifier on the frozen representations. As shown in Table 3, CML achieves 87.0% (X-Sub) and 93.9% (X-View) accuracy on NTU-60 and 79.2% (X-Sub) with 79.8% (X-Set) accuracy on NTU-120. Compared to supervised ST-GCN [32], CML demonstrates significant improvements: 5.5% and 5.6% on NTU-60 X-Sub and X-View tasks, and 8.5% and 6.7% on NTU-120 X-Sub and X-Set tasks, respectively. These results not only surpass the performance of supervised ST-GCN [32] but also validate the effectiveness of our self-supervised pre-training approach.

### 4.4. Ablation Study

**Benefit of Masked Skeleton Modeling**: To verify the effectiveness of masked skeleton learning, we pre-trained the CML (w/o. MSL) model, which has no reconstruction branch and the same basic settings as CML. The performance of CML and CML (w/o. MSL) is compared in Table 4 on NTU-60 X-View tasks. Specifically, CML demonstrates improved representation learning performance by 4.2% compared with CML (w/o. MSL), which shows that sole contrastive learning fails to achieve high performance, and our contrastive mask learning scheme does boost representation learning.

**Benefit of Multi-Level Contrast**: To verify the effectiveness of multi-level contrastive learning, we pre-trained the CML (w/o. ins) and CML (w/o. clu) models in order to remove instance-level contrast and cluster-level contrast, respectively, while basic settings were the same as those of CML (w/o. MSL). The performance of CML, CML (w/o. ins), and CML (w/o. clu) on NTU-60 X-View tasks is compared in Table 5. Specifically, CML demonstrates improved recognition accuracy by 6.0% and 7.6% compared with CML (w/o. ins) and CML (w/o. clu), which shows that single-level contrastive learning fails to achieve high performance, and our contrastive mask learning scheme does boost representation learning.

**Benefit of Mutual Learning**: To determine how the mutual learning strategy affects the performance, the CML (w/o. ML) model with no extra benefit from the construction branch was used in cluster contrast, and the parameters of the construction branch were not updated by the student branch. Other basic settings were identical to those of CML, and the results are shown in Table 6. To be specific, the mutual learning strategy demonstrates improved performance by 3.1% on NTU-60 X-View tasks, which demonstrates that the mutual learning strategy is beneficial to the contrastive mask learning scheme.

### 4.5. Visualization Results

**Figure 2:**
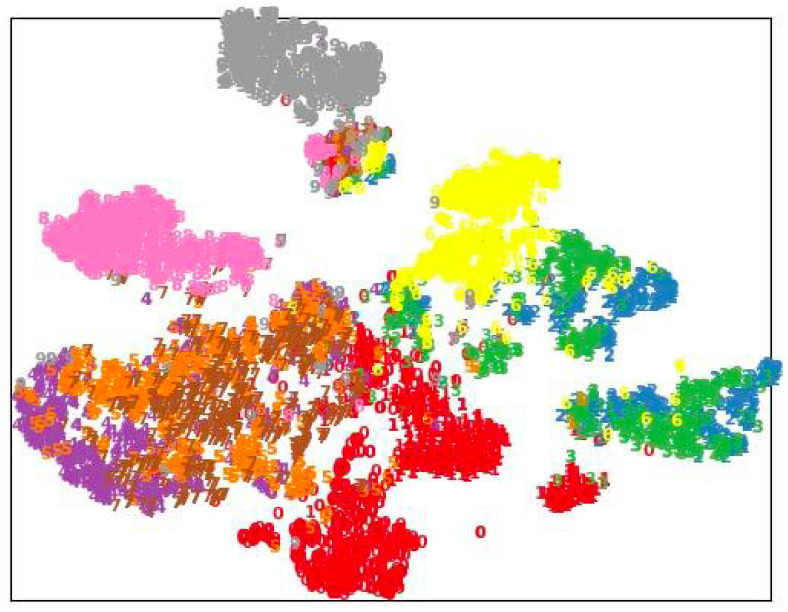
t-SNE visualization of embedding for AML [14] on the NTU-60 X-View task (best viewed in color).

**Figure 3:**
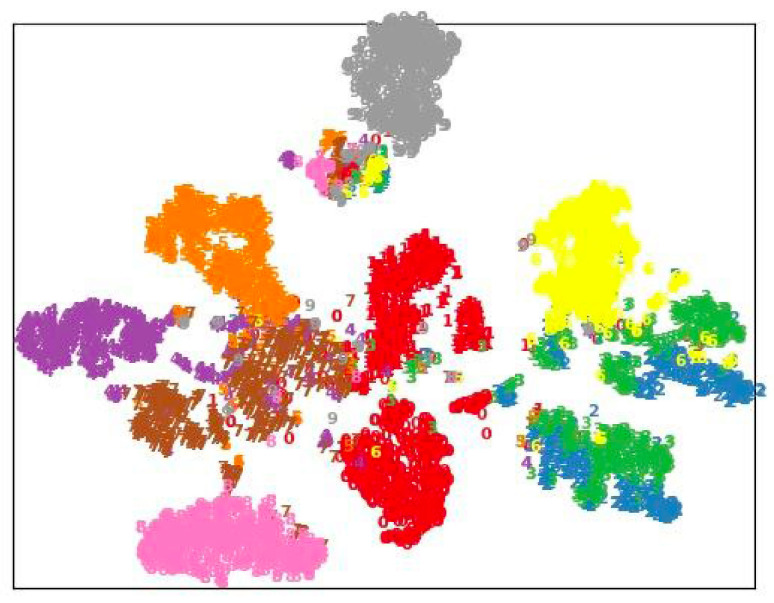
t-SNE visualization of embedding for CML on the NTU-60 X-View task (best viewed in color).

As illustrated in Figure 2 and Figure 3, we leveraged t-SNE to visualize the embedding clustering produced by CML and AML [14]. Note that the embedding of 10 different action categories was sampled and visualized with different colors. The visual results show how the embedding of the same class of actions forms clusters, while different classes of actions are separated. By comparing the t-SNE of CML and AML [14], it can be seen that CML improves the clustering of actions, which indicates that the learned latent space is more discriminative.

## 5. Conclusions

In this study, a novel self-supervised learning approach referred to as a contrastive mask learning scheme (CML) was developed for 3D skeleton action representation learning. By injecting the mask learning mechanism into multi-level contrastive learning with a mutually beneficial learning strategy, we managed to integrate contrastive learning and masked skeleton modeling comprehensively and enable intra-/inter-instance consistency pursuit via multi-level contrast, which led to more discriminative skeleton representation learning. The experimental results validated that the proposed CML outperforms existing state-of-the-art methods on two challenging datasets: NTU RGB+D and PKU-MMD.

## Figures and Tables

**Table 1:** Comparison of our model with existing methods on NTU and PKU-MMD.

Architecture	NTU-60 (%)		NTU-120 (%)		PKU-MMD (%)	
	X-Sub	X-View	X-Sub	X-Set	Part I	Part II
*Supervised*						
C-CNN + MTLN [37]	79.6	84.8	-	-	-	-
TSRJI [38]	73.3	80.3	67.9	62.8	-	-
ST-GCN [32]	81.5	88.3	70.7	73.2	84.1	48.2
*Self-supervised*						
LongT GAN [6]	39.1	48.1	-	-	67.7	27.0
ASCAL [8]	58.5	64.8	48.6	49.2	-	-
MS2L [7]	52.6	-	-	-	64.9	27.6
P&C [9]	50.7	76.3	-	-	-	-
ISC [27]	76.3	85.2	67.9	67.1	80.9	36.0
CrosSCLR (3S) [10]	77.8	83.4	67.9	66.7	84.9	-
CPM (3S) [39]	83.2	87.0	73.0	74.0	90.7	51.5
CSCLR (3S) [29]	80.1	85.2	69.2	70.2	89.3	45.1
HiCo [30]	81.4	88.6	73.7	74.5	89.4	54.7
ViA [13]	78.1	85.8	69.2	66.9	-	-
AML(3S) [14]	84.9	89.7	75.3	77.3	93.2	58.0
CML (3S)	86.8	91.1	77.5	78.9	93.5	58.6

**Table 2:** Semi-supervised results and comparison with the existing methods on NTU-60.

Architecture	Label Fraction (%)	X-Sub (%)	X-View (%)
ISC [27]	1	35.7	38.1
SRCL [39]	1	50.7	49.7
HiCo [30]	1	54.4	54.8
CPM [28]	1	56.7	57.5
CSCLR [29]	1	55.4	57.1
AML [14]	1	58.9	59.7
CML	1	59.1	60.2
ISC [27]	10	65.9	72.5
SRCL [39]	10	69.3	73.6
CPM [28]	10	73.0	77.1
HiCo [30]	10	73.0	78.3
CSCLR [29]	10	78.6	81.8
AML [14]	10	79.0	82.3
CML	10	80.1	83.4

**Table 3:** Fine-tuned results and comparison with existing methods on NTU-60/120.

Architecture	NTU-60 (%)		NTU-120 (%)	
	X-Sub	X-View	X-Sub	X-Set
C-CNN + MTLN [37]	79.6	84.8	-	-
TSRJI [38]	73.3	80.3	67.9	62.8
ST-GCN [32]	81.5	88.3	70.7	73.2
CML	87.0	93.9	79.2	79.8

**Table 4:** Benefit of masked skeleton modeling on NTU-60 X-View.

Architecture	X-View (%)
CML (w/o. MSL)	83.4
CML	87.6

**Table 5:** Benefit of multi-level contrast on NTU-60 X-View.

Architecture	X-View (%)
CML (w/o. ins)	77.4
CML (w/o. clu)	75.8
CML	83.4

**Table 6:** Benefit of mutual learning strategy on NTU-60 X-View.

Architecture	X-View (%)
CML (w/o. ML)	80.3
CML	83.4

## Data Availability

The datasets generated during and/or analyzed during the current study are available from the corresponding author upon reasonable request.
